# Assessment of Possible Contributions of Hyaluronan and Proteoglycan Binding Link Protein 4 to Differential Perineuronal Net Formation at the Calyx of Held

**DOI:** 10.3389/fcell.2021.730550

**Published:** 2021-09-17

**Authors:** Kojiro Nojima, Haruko Miyazaki, Tetsuya Hori, Lydia Vargova, Toshitaka Oohashi

**Affiliations:** ^1^Department of Molecular Biology and Biochemistry, Okayama University Graduate School of Medicine, Dentistry and Pharmaceutical Sciences, Okayama, Japan; ^2^Cellular and Molecular Synaptic Function Unit, Okinawa Institute of Science and Technology Graduate University, Okinawa, Japan; ^3^Department of Neuroscience, Charles University, Second Faculty of Medicine, Prague, Czechia; ^4^Department of Cellular Physiology, Institute of Experimental Medicine AS CR, Prague, Czechia

**Keywords:** perineuronal net, chondroitin sulfate proteoglycan, calyx of Held, hyaluronan and proteoglycan binding link protein 4, synapse, *in situ* proximity ligation assay

## Abstract

The calyx of Held is a giant nerve terminal mediating high-frequency excitatory input to principal cells of the medial nucleus of the trapezoid body (MNTB). MNTB principal neurons are enwrapped by densely organized extracellular matrix structures, known as perineuronal nets (PNNs). Emerging evidence indicates the importance of PNNs in synaptic transmission at the calyx of Held. Previously, a unique differential expression of aggrecan and brevican has been reported at this calyceal synapse. However, the role of hyaluronan and proteoglycan binding link proteins (HAPLNs) in PNN formation and synaptic transmission at this synapse remains elusive. This study aimed to assess immunohistochemical evidence for the effect of HAPLN4 on differential PNN formation at the calyx of Held. Genetic deletion of *Hapln4* exhibited a clear ectopic shift of brevican localization from the perisynaptic space between the calyx of Held terminals and principal neurons to the neuropil surrounding the whole calyx of Held terminals. In contrast, aggrecan expression showed a consistent localization at the surrounding neuropil, together with HAPLN1 and tenascin-R, in both gene knockout (KO) and wild-type (WT) mice. An *in situ* proximity ligation assay demonstrated the molecular association of brevican with HAPLN4 in WT and HAPLN1 in gene KO mice. Further elucidation of the roles of HAPLN4 may highlight the developmental and physiological importance of PNN formation in the calyx of Held.

## Introduction

Perineuronal nets (PNNs) are pericellular coats of condensed matrix that enwrap the cell bodies and dendrites of certain neurons in the adult central nervous system. In the brain, PNNs are completed at the end of developmental critical periods for experience-dependent plasticity. They contribute to the stabilization of specific connection patterns, thus limiting plasticity. More recently, PNNs have been revealed to have myriad actions in many central nervous system functions, including memory, psychiatric disease, and neurodegeneration (Fawcett et al., 2019; Carulli and Verhaagen, 2021). PNNs primarily consist of hyaluronan, chondroitin sulfate proteoglycans (CSPGs) of the lectican family, tenascin-R, and link proteins as a core extracellular matrix (ECM). Other ECM-affiliated molecules (such as semaphorin 3A) and ECM regulators (such as a disintegrin and metalloproteinase with thrombospondin motifs (ADAMTS) proteases) are also included. The heterogeneity of PNNs may arise from variations in molecular compositions as well as differences in the glycan structure of CSPGs.

Perineuronal nets are typically found around fast-spiking GABAergic interneurons expressing parvalbumin. However, of note is that they also exist surrounding other neurons, such as the medial nucleus of the trapezoid body (MNTB). Each principal cell within the MNTB is contacted by a single giant terminal called the calyx of Held, which is characterized by fast and highly reliable synaptic transmission (Borst and Soria van Hoeve, 2012; Joris and Trussell, 2018; Nakakubo et al., 2020). In addition, a unique and clearly distinct distribution pattern of the proteoglycans aggrecan and brevican on the surface of the MNTB principal neurons/calyx of Held has been reported (Blosa et al., 2013). Electrophysiological studies on genetic models deficient in PNN components or enzymatic chondroitin sulfate chain depletion models have highlighted the importance of PNN components and their physiological functions in the calyx of Held (Blosa et al., 2015; Balmer, 2016; Schmidt et al., 2020).

The hyaluronan and proteoglycan binding link proteins (HAPLNs) are key molecules in the formation and control of hyaluronan-based condensed perineuronal matrix in the adult brain (Carulli et al., 2010; Kwok et al., 2010; Bekku et al., 2012; Fawcett et al., 2019). *Hapln4/Bral2*-knockout (KO) mice have attenuated PNNs mainly expressed in the brainstem and cerebellum (Bekku et al., 2012). Moreover, the loss of HAPLN4 markedly affected the localization of brevican in all of the nuclei examined, whereas no effect was seen on aggrecan localization (Bekku et al., 2012). HAPLN4 is typically expressed in auditory brainstem neurons, including MNTB neurons (Bekku et al., 2003). Studies in *Hapln4*-KO mice detected higher hearing thresholds at high frequencies and weaker temporal resolution ability in KO mice than in wild-type (WT) mice (Popelář et al., 2017). We have also reported the effect of HAPLN4 deficiency on extracellular diffusion parameters in MNTB during aging (Sucha et al., 2020). These results suggest the importance of HAPLN4 in auditory function. Furthermore, *Hapln4*-KO mice have demonstrated that HAPLN4 is a selective regulator for the formation and transmission of GABAergic Purkinje synapses and deep cerebellar nuclei neurons (Edamatsu et al., 2018). These findings suggest that HAPLNs may regulate the micro-organization of PNN *via* specific interactions with lecticans (HAPLN4 with brevican and HAPLN1 with aggrecan) (Oohashi et al., 2015).

Previous electron microscopic investigations delineated the precise distribution pattern of aggrecan and brevican at the calyx of Held (Blosa et al., 2013), namely: brevican prominently localizes to the pericellular space between the calyx of Held terminals and principal neurons to seal the synaptic cleft. In contrast, aggrecan encloses the entire calyx of Held terminals and principal cells. The differential PNN formation of aggrecan and brevican might indicate their distinct functions on the cell/synapse surface.

These findings led us to conclude that different HAPLN–lectican molecular sets may orchestrate distinct PNNs with functional relevance. To verify this hypothesis, we evaluated the effect of the genetic deletion of *Hapln4* on the differential formation of PNNs using immunohistochemistry and *in situ* proximity ligation assay (PLA) in *Hapln4*-KO mice compared with WT mice.

## Materials and Methods

### Animals

Homozygous *Hapln4*-KO mice aged 4 to 5 months and age-matched WT animals from a C57Bl/6 background were used in the experiments. The KO mice were generated by homologous recombination in embryonic stem cells, as described previously (Bekku et al., 2012). The animals were kept on a 12-h light/dark cycle, with a regular feeding and cage cleaning schedule. The mice were given free access to food and water. This study was conducted in strict accordance with the Policy on the Care and Use of Laboratory Animals, Okayama University. The protocol was approved by the Animal Care and Use Committee of the Okayama University (protocol number: OKU-2020743).

### Immunohistochemistry and *in situ* Proximity Ligation Assay

Immunohistochemistry was performed as previously described (Cicanic et al., 2018; Edamatsu et al., 2018). The animals were deeply anesthetized with isoflurane inhalation. To obtain specimens for cryosections, vascular perfusion *via* the left ventricle was performed with phosphate-buffered saline (PBS) and then with a fixative agent containing 4% paraformaldehyde in 0.1 M phosphate buffer (pH 7.4). The brains were removed and postfixed overnight at 4°C. The samples were immersed in 30% sucrose solution in PBS at 4°C, embedded in optimal cutting temperature compound (Sakura Finetek, Japan), and frozen. Then, 30 μm-thick coronal cryosections were cut on a cryostat (Leica CM 1,860) and further processed.

The sections were permeabilized with 0.2% Triton X-100 in PBS and then blocked in 10% donkey serum (Sigma, St. Louis, MO, United States; D9663) in PBS or a Duolink^TM^ blocking solution. Regarding aggrecan immunolabeling, pretreatment with chondroitinase ABC (ChABC, 0.1 U/ml; Sigma, C2905) for 40 min at 37°C was required. The specimens were incubated overnight at 4°C with specific primary antibodies diluted in PBS with 0.2% Triton X-100 and 1.5% donkey serum. Then, the slices were incubated for 4 h at room temperature with secondary antibodies. The following primary antibodies were used: goat anti-HAPLN4 polyclonal antibody (R&D Systems, Minneapolis, MN, United States; AF4085, RRID:AB_2116264; dilution 1:50), goat anti-HAPLN1 polyclonal antibody (R&D Systems; AF2608, RRID:AB_2116135; dilution 1:50), rabbit anti-aggrecan polyclonal antibody (Merk Millipore, Burlington, MA, United States; AB1031, RRID:AB_90460; dilution 1:150), rabbit anti-brevican polyclonal antibody ([+1058], Thon et al., 2000; dilution 1:2,000; kindly gifted by Dr. Takako Sasaki, Oita University), goat anti-tenascin-R polyclonal antibody (R&D Systems; AF3865, RRID:2207009; dilution 1:200), and guinea pig anti-vesicular glutamate transporter 1 (VGLUT1) polyclonal antibody (Synaptic systems, Göttingen, Germany, 135 304, RRID:AB_887878; dilution 1:100). Regarding immunohistochemistry, the following secondary antibodies were used: Alexa 488-conjugated donkey anti-goat IgG (Thermo Fisher Scientific, Tokyo, Japan; A11055, RRID:AB_2534102; dilution 1:400), Alexa 594-conjugated donkey anti-guinea pig IgG (Jackson ImmunoResearch, West Grove, PA, United States; 706-586-148, RRID:AB_2340475; dilution 1:400), and Alexa 647-conjugated donkey anti-rabbit IgG (Abcam, Cambridge, United Kingdom, ab150075, RRID:AB_2752244; dilution 1:400).

*In situ* PLA was conducted according to the instructions of the manufacturer (Sigma, Duolink^®^). After primary antibody incubation, the secondary antibodies conjugated with oligonucleotides (PLA probe MINUS and PLA probe PLUS) were added to the specimens and incubated for 2 h at room temperature. Further ligation and amplification reactions were performed. The ligation solution, consisting of two oligonucleotides and ligases, was added and incubated for 30 min at 37°C to cause the oligonucleotides to hybridize to the two PLA probes and join a closed circle when they are in close proximity (<40 nm apart). In the final rolling-circle amplification reaction, the amplification–polymerase solution was added and incubated for 100 min at 37°C. The control experiments were performed for each combination of PLA probes by omitting one of the primary antibodies.

A biotinylated HA-binding protein (b-HABP: Hokudo, Sapporo, Japan, BC41) derived from versican using recombinant human G1 domain was used for hyaluronan staining, as described previously (Bekku et al., 2010). Sections were incubated at 4°C overnight with b-HABP (dilution 1:100), followed by secondary labeling with Alexa 488-conjugated streptavidin (Thermo Fisher Scientific, S32354; dilution 1:400).

After tissue processing, the sections were mounted on microscope slides with a fluorescence mounting medium (Dako, S3023) or Duolink^®^ *in situ* Mounting Medium with DAPI (Sigma). Fluorescent images were acquired using a confocal laser scanning microscope system (Carl Zeiss, LSM780). Confocal images were acquired at a resolution of 1,024 × 1,024 dpi. Laser intensity, gain, and offset were maintained at constant levels for each analysis. Staining intensities were analyzed using the plot profile tool in ImageJ FIJI software. Each experiment was repeated using three WT and three *Hapln4*-KO mice. All experiments were successively repeated at least two times in each mouse, and similar results were obtained. Hence, a representative result is shown in the figure.

## Results

### Distribution of Hyaluronan and Proteoglycan Binding Link Protein 4 and Related Molecules in the Medial Nucleus of the Trapezoid Body

In a previous report, two distinct PNN-type proteoglycan expressions have been reported in the MNTB (Blosa et al., 2013), namely, brevican localization at the perisynaptic space between the calyx of Held terminals and principal neurons and aggrecan localization at the surrounding neuropil. To compare the precise distribution of HAPLN4 with other related molecules and confirm its localization in the context of these distinct PNNs, we performed immunohistochemistry.

**FIGURE 1:**
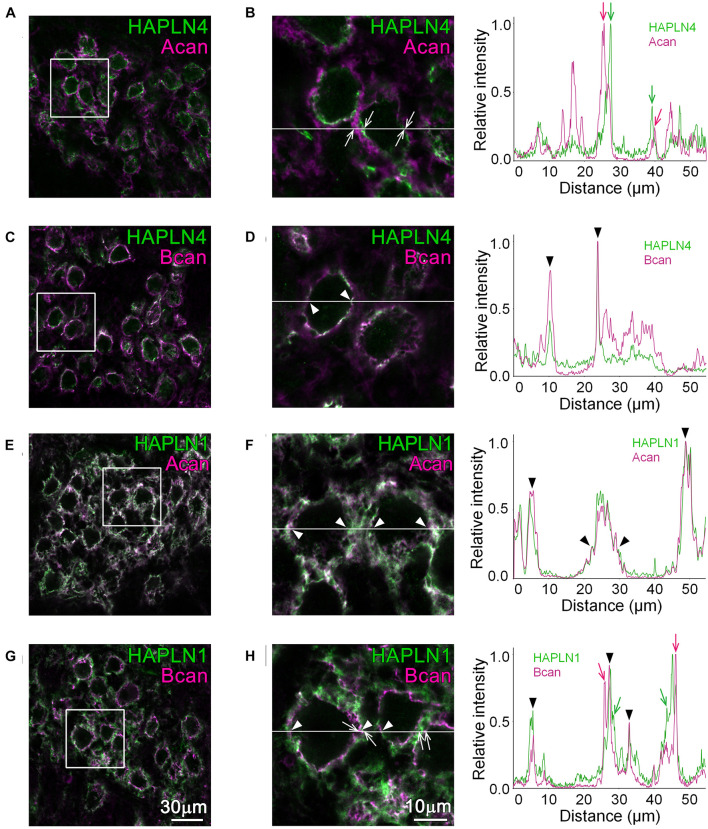
Distribution of HAPLNs and lecticans in the medial nucleus of the trapezoid body (MNTB) of adult wild-type (WT) mice. Immunohistochemical detection of HAPLNs (green) and lecticans (magenta) in the MNTB of 4-month-old adult WT mice using HAPLN4 **(A–D)**, HAPLN1 **(E–H)**, aggrecan **(A,B,E,F)**, and brevican **(C,D,G,H)** immunostaining. The intensity profiles of the fluorescence signals along the white lines are shown in the rightmost panels. The fluorescence intensities are normalized by the highest intensities of individual proteins within the lines. Of note are the well-segregated signals between HAPLNs and CSPGs that are indicated by arrows (HAPLNs in green and CSPGs in magenta, respectively). The colocalized signals are indicated by arrowheads. Acan, aggrecan; Bcan, brevican. Scale bar = 30 μm in the left panels and 10 μm in the right panels.

In the MNTB of WT, linear immunolabeling of HAPLN4, which colocalized with that of brevican, was observed (Figures 1C,D; see arrowheads in the right panels), while a clear segregation of HAPLN4 and aggrecan immunoreactivity (Figures 1A,B; see arrows in the right panels) was noted. On the other hand, HAPLN1 immunostaining overlapped with that of aggrecan in the extracellular space of principal cells (Figures 1E,F; see arrowheads in the right panels). Notably, HAPLN1 immunoreactivity enwrapped the inner ring-like brevican immunostaining in stark contrast (Figures 1G,H; see arrowheads in the right panels). Furthermore, by fluorescence intensity profile analysis, we noticed that some weak staining of brevican localized to the surrounding extracellular space partially overlapped with that of HAPLN1, which indicates a potential interaction between brevican and HAPLN1 (Figure 1H; see arrowheads in the right panels).

### Ectopic Expression of Brevican in the Medial Nucleus of the Trapezoid Body of *Hyaluronan and Proteoglycan Binding Link Protein 4*-Knockout Mice

**FIGURE 2:**
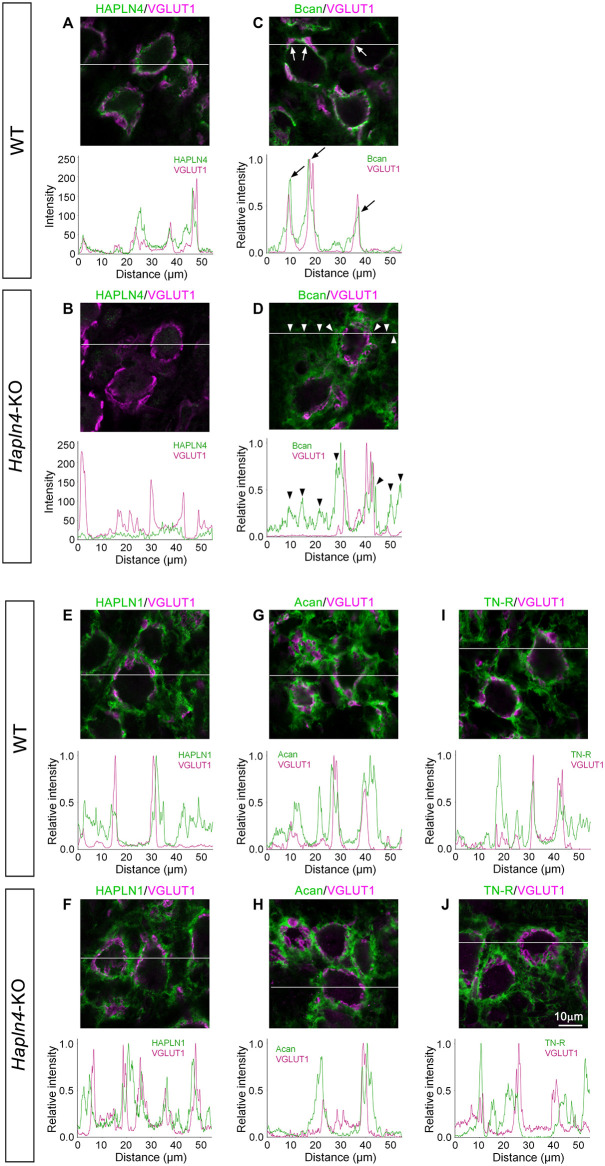
Ectopic expression of brevican in the medial nucleus of the trapezoid body (MNTB) of *Hapln4*-KO mice. To clarify the spatial location of perineuronal net components, the immunoreactivity of HAPLNs **(A,B,E,F)**, brevican or aggrecan **(C,D,G,H)**, and tenascin-R **(I,J)** (green) is examined in the MNTB of 4-month-old adult mice using VGLUT1 as a marker of the calyces of Held (magenta). The intensity profiles of the fluorescence signals along the white lines are shown in the lower panels. The fluorescence intensities are normalized by the highest intensities of individual proteins within the lines except in **(A,B)**. Since these have a relatively low signal-to-noise ratio of the anti-HAPLN4 antibodies, the background is high when the intensities are normalized as previously described. Of note is the observed ectopic shift of brevican immunolocalization from the perisynaptic space in the wild type (arrows) to the surrounding extracellular space in the neuropil in *Hapln4*-KO (arrowheads). Acan, aggrecan; Bcan, brevican; TN-R, tenascin-R. The scale bar represents 10 μm.

In our previous reports, the diffuse expression of brevican in the MNTB of the young adult *Hapln4*-KO (aged 2–4 months) has been reported (Bekku et al., 2012; Sucha et al., 2020). However, the detailed spatial relationship of PNN components around the MNTB principal cells is lacking. To clarify the special location of PNN components, giant glutamatergic synaptic terminals, and the calyces of Held, were visualized as histological markers by immunostaining for VGLUT1 (Blosa et al., 2013). Regarding MNTB in WT, linear immunostaining for HAPLN4 and brevican around each principal cell was surrounded by VGLUT1 staining (Figures 2A,C). In contrast, the immunoreaction of brevican in *Hapln4*-KO mice was prominently located around VGLUT1 staining (Figure 2D). This clearly demonstrates the ectopic shift of brevican localization at the surrounding extracellular space in the neuropil in the absence of HAPLN4 (Figure 2B). Moreover, we found another interesting aspect of HAPLN1-based PNN in MNTB. Immunostaining for HAPLN1 and aggrecan was consistently localized at the surrounding extracellular space in both genotypes (Figures 2E–H). To assess the relative localization of HAPLN1 and brevican in *Hapln4*-KO, we compared the immunolocalization of these molecules and VGLUT1 by triple immunostaining in the same section (Supplementary Figure 1). The fluorescence intensity profile analysis showed the well-matched colocalization of HAPLN1 and brevican at the surrounding extracellular space in the neuropil in *Hapln4*-KO. In addition, we assessed the localization of tenascin-R, another important crosslinker of lecticans, in the PNN. The immunoreactivity of tenascin-R was prominent at the HAPLN1-positive surrounding extracellular space around VGLUT1 staining. However, it was barely detectable at the inner ring-like extracellular space between the calyx of Held terminals and the principal cells in both genotypes (Figures 2I,J). Hyaluronan labeling with b-HABP showed two distinct PNNs in both genotypes, namely, the inner ring-like PNNs in the perisynaptic space between the calyx of Held terminals and principal neurons and the PNNs at the surrounding extracellular space with neuropil-like morphology (Supplementary Figure 2).

### Importance of Specific *Hyaluronan and Proteoglycan Binding Link Protein*–Lectican Molecular Interactions for Distinct Perineuronal Net Formation at the Calyx of Held

**FIGURE 3:**
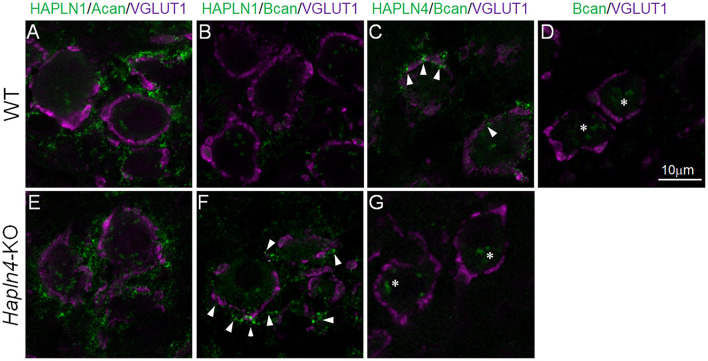
Detection of specific HAPLN–lectican molecular interactions by an *in situ* proximity ligation assay (PLA). *In situ* PLA to visualize *in situ* endogenous HAPLN–lectican interactions (green) in the medial nucleus of the trapezoid body of 5-month-old adult WT **(A–D)** and KO **(E–G)** mice using VGLUT1 as a marker of the calyx of Held (magenta). A pair of primary antibodies of HAPLN4 and brevican shows dot signals at the edge or perisynaptic space of the calyx of Held terminals (**C**, arrowheads). Notably, in the absence of HAPLN4, brevican shows an ectopic shift of localization to the surrounding neuropil, where HAPLN1 might potentially stabilize brevican in the aberrant condition (**F**, arrowheads). The specificity of the PLA reaction is validated with two different negative controls: one is a reaction that omits the anti-HAPLN4 antibody on wild-type tissue **(D)**; the other is the PLA reaction of HAPLN4 and brevican using *Hapln4*-KO tissue **(G)**. The non-specific reactions in the negative controls are indicated by asterisks **(D,G)**. Acan, aggrecan; Bcan, brevican. The scale bar represents 10 μm.

To better understand the molecular mechanism for the ectopic expression of brevican in the MNTB of *Hapln4*-KO, we introduced *in situ* PLA, a method that outperforms the *in situ* visualization of endogenous protein–protein interactions in tissue sections. By combining immunostaining for the caliceal marker VGLUT1, it becomes a powerful approach to identify differential interactions between HAPLNs and lecticans. The presence of PLA-positive green dots indicates the existence of HAPLN/lectican complexes (Figure 3).

A pair of primary antibodies of HAPLN1 and aggrecan generated abundant signals at the surrounding extracellular space with neuropil-like morphology around VGLUT1 staining in both WT and KO mice (Figures 3A,E). In contrast, another pair of primary antibodies of HAPLN4 and brevican could generate dot signals at the edge or perisynaptic space of the calyx of Held terminals (Figure 3C, arrowheads) in WT mice. An aberrant pair of primary antibodies of HAPLN1 and brevican showed prominent signals at the surrounding extracellular space in *Hapln4*-KO mice (Figure 3F, arrowheads), which were very similar to those between HAPLN1 and aggrecan interactions (Figures 3A,E), while this pair produced scarcely detectable signals in WT mice (Figure 3B).

**FIGURE 4:**
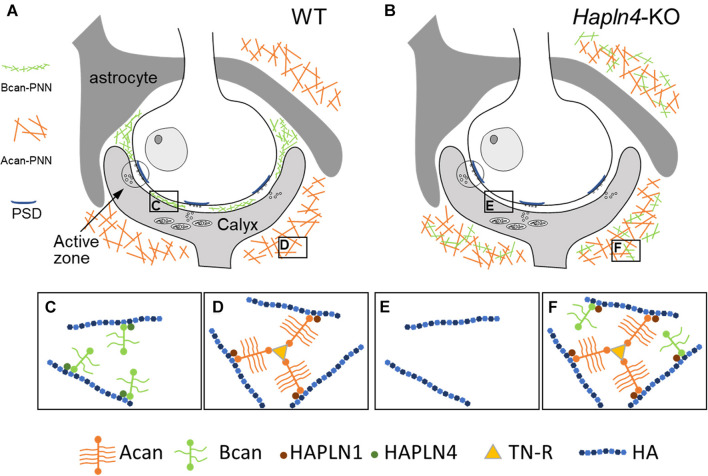
Schematic representation of HAPLN-dependent perineuronal net (PNN) micro-organization at the calyx of Held synapses. **(A,C,D)** In wild-type mice, HAPLN4-brevican-type PNN is enriched in the perisynaptic space between the calyx of Held terminals and the principal neuron, and HAPLN1–aggrecan–tenascin-R-type PNN surrounds the whole calyx of Held terminals. **(B,E,F)** In *Hapln4*-KO, the ectopic expression of brevican is detectable with HAPLN1 association at the surrounding extracellular space in the neuropil in addition to the HAPLN1–aggrecan–tenascin-R complex. Acan, aggrecan; Bcan, brevican; TN-R, tenascin-R; HA, hyaluronan; PSD, postsynaptic density.

This study provides direct evidence for differential interactions between HAPLNs and lecticans. In WT form, a specific combination of HAPLN and lectican is located at the specific extracellular milieu, in which HAPLN4 and brevican are located in the perisynaptic space near the synaptic cleft, HAPLN1, and aggrecan surrounding the whole calyx of Held terminals (Figures 4A,C,D). Notably, in the absence of HAPLN4, brevican showed an ectopic shift of localization to the surrounding neuropil, where HAPLN1 could potentially stabilize brevican in the aberrant condition (Figures 4B,E,F).

## Discussion

In this study, we hypothesized that distinct HAPLNs would regulate the micro-organization of PNN *via* specific interactions with lecticans (Oohashi et al., 2015). Our results demonstrated that HAPLN4 is important for maintaining the specific localization of brevican in the perisynaptic space between the calyx of Held terminals and the principal neurons in the MNTB.

A query could be raised about how each HAPLN-dependent microorganization would be distinctively formed. The authors assumed that some of the following requirements were necessary to achieve this: (i) differences in binding affinity between HAPLNs and G1 domain of lecticans, (ii) tenascin-R crosslinking of aggrecan *via* the G3 domain, and (iii) HAPLN4 translocation through the axon and secretion from the calyx of Held terminal. Regarding the first requirement, we demonstrated a clear difference in HAPLN and lectican binding between WT and KO mice using *in situ* PLA (Figure 3). However, biochemical experiments are necessary to measure the binding affinity between HAPLNs and lecticans. Regarding the second requirement, tenascin-R has been shown to have a high affinity for the G3 domain of aggrecan and brevican (Aspberg, 2012). Moreover, it has been experimentally demonstrated that tenascin-R could promote the assembly of reticular PNNs *via* cross-linking of aggrecan (Morawski et al., 2014). In fact, tenascin-R was prominently expressed to surround the entire calyx of Held terminals (Figure 2). The cross-linking of aggrecan *via* tenascin-R in HAPLN1-based PNN may increase the structural integrity of the PNN. The third requirement has not been validated yet. However, there have been several supporting data from three previous reports that may explain the possibility of HAPLN4 axonal transport (Bekku et al., 2003; Carulli et al., 2007; Blosa et al., 2016). Further research is necessary to test this hypothesis.

Each principal neuron in the MNTB receives a single input from a giant axosomatic terminal: the calyx of Held. High transmission reliability and consistency in timing and amplitude have been confirmed in the mature calyx of Held synapses (Sonntag et al., 2011; Borst and Soria van Hoeve, 2012). Although the calyx of Held synapses are enwrapped by densely organized PNNs, the functional importance of PNN has not been elucidated until recently. However, based on the recent progress in understanding the formation of PNN (Sonntag et al., 2015), Morawski et al. (2014) have addressed the functional role of PNN components. They could take advantage of the accessibility of the calyx of Held synapses to patch-clamp recordings in brain slices from a genetic model deficient in PNN components (Blosa et al., 2015; Schmidt et al., 2020). In brevican-deficient mice, the speed of pre- to post-synaptic action potential transmission was reduced, and the duration of the respective pre- and post-synaptic action potentials increased (Blosa et al., 2015). A significant prolongation of transmission speed at the calyx of Held has been reported in neurocan-deficient mice (Schmidt et al., 2020).

Perineuronal nets have been implicated in a number of psychiatric disorders (Fawcett et al., 2019). Recently, the *HAPLN4* gene has been included as a significant gene according to the transcriptome-wide association study FUSION in its analysis of 13,435 genes using gene expression data from the PsychENCODE Consortium (1,321 brain samples) (Gandal et al., 2018; Mullins et al., 2021). Furthermore, the Schizophrenia Working Group of the Psychiatric Genomics Consortium identified 108 schizophrenia-related loci (Schizophrenia Working Group of the Psychiatric Genomics Consortium, 2014). Although gray matter volume (GMV) reduction is a common neuroimaging finding of schizophrenia, little is known about the underlying mechanism for the GMV reduction. Ji et al. (2021) applied transcription (196 schizophrenia risk genes)—a neuroimaging analysis to test which of these genes were associated with GMV alterations in patients with schizophrenia. Finally, they identified 98 genes associated with GMV alterations in patients with schizophrenia. Of note is that the 98 identified genes showed a significant enrichment on eight gene ontology terms for molecular functions, including CSPG binding and hyaluronan binding. Among them, *HAPLN4* showed the largest negative correlation: the expression of this gene was lower in the brain regions with more GMV reduction in patients with schizophrenia. The authors suggested that the low expression level of *HAPLN4* might lead to GMV reduction by influencing the ECM and PNNs. This result is in line with our previous findings that HAPLN4 deficiency in mice led to a decrease in the extracellular space volume fraction but only in the aged brain (Cicanic et al., 2018; Sucha et al., 2020).

Hyaluronan and proteoglycan binding link proteins play an important role as organizers of PNNs. Moreover, the current study indicated that each HAPLN may contribute to the formation of distinct PNNs with different functional relevance. The main limitation to this study is the lack of functional studies on aberrant PNN at the calyx of Held in *Hapln4*-KO mice. Regarding the direct evaluation of the developmental and physiological role of HAPLN4, electrophysiological studies on the calyx of Held–MNTB synapses in *Hapln4*-KO would be a powerful approach.

## Conclusion

Our results demonstrated a clear ectopic shift of brevican localization from the perisynaptic space between the calyx of Held terminals and principal neurons to the surrounding neuropil. In contrast, aggrecan expression showed a consistent localization at the surrounding neuropil together with HAPLN1 and tenascin-R in both KO and WT mice.

## Data Availability Statement

The original contributions presented in the study are included in the article/Supplementary Material, further inquiries can be directed to the corresponding author.

## Ethics Statement

The animal study was reviewed and approved by the Animal Care and Use Committee of Okayama University.

## Author Contributions

TO, TH, and LV contributed to the study concept and design. KN and HM contributed to the performance of the experiments. KN, HM, TH, LV, and TO contributed to the analysis and interpretation of data. TO, HM, and KN prepared the manuscript initial draft. All authors participated in the critical correction of the manuscript and approved the final version.

## Conflict of Interest

The authors declare that the research was conducted in the absence of any commercial or financial relationships that could be construed as a potential conflict of interest.

## Publisher’s Note

All claims expressed in this article are solely those of the authors and do not necessarily represent those of their affiliated organizations, or those of the publisher, the editors and the reviewers. Any product that may be evaluated in this article, or claim that may be made by its manufacturer, is not guaranteed or endorsed by the publisher.
