# Supplementary material for: Assessment of Possible Contributions of Hyaluronan and Proteoglycan Binding Link Protein 4 to Differential Perineuronal Net Formation at the Calyx of Held
Source: Front Cell Dev Biol. 2021 Sep 17;9:730550. doi: 10.3389/fcell.2021.730550 (PMC8485899; doi:10.3389/fcell.2021.730550)
Supplement: Supplementary file 1 [file Data_Sheet_1.pdf]

Supplementary Material

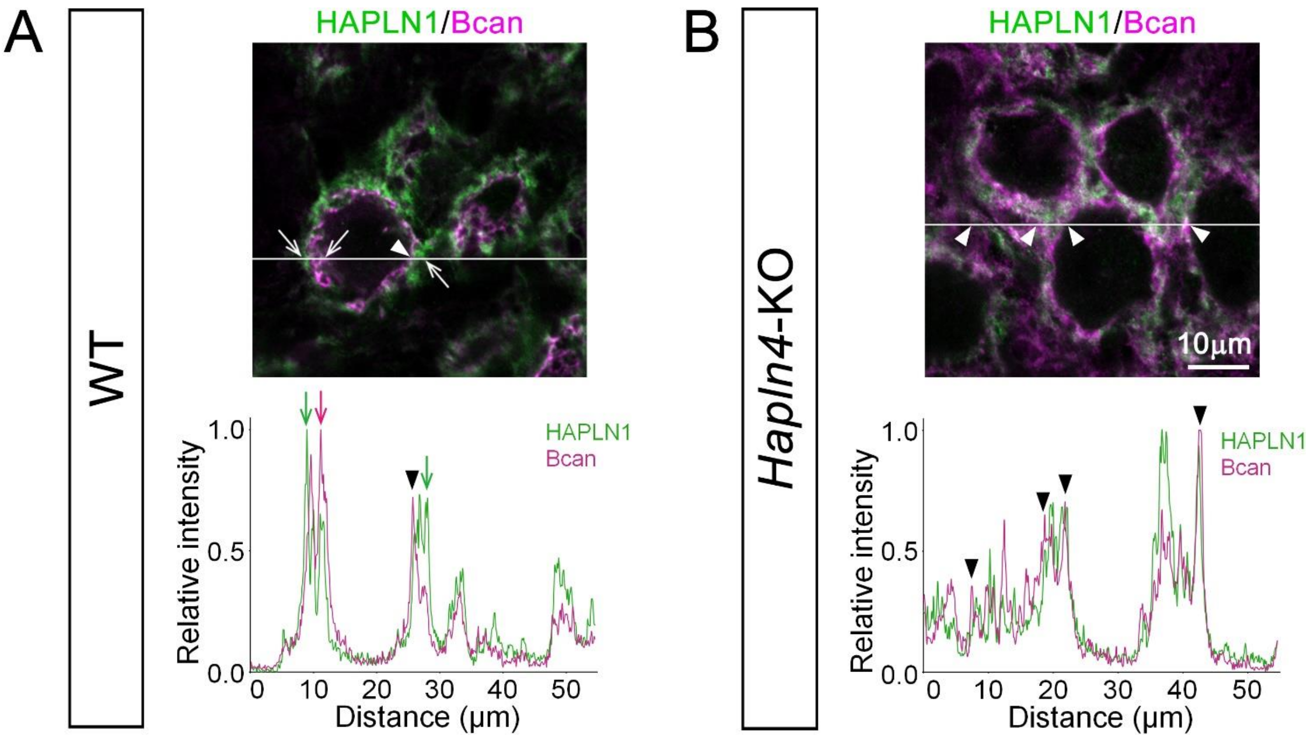

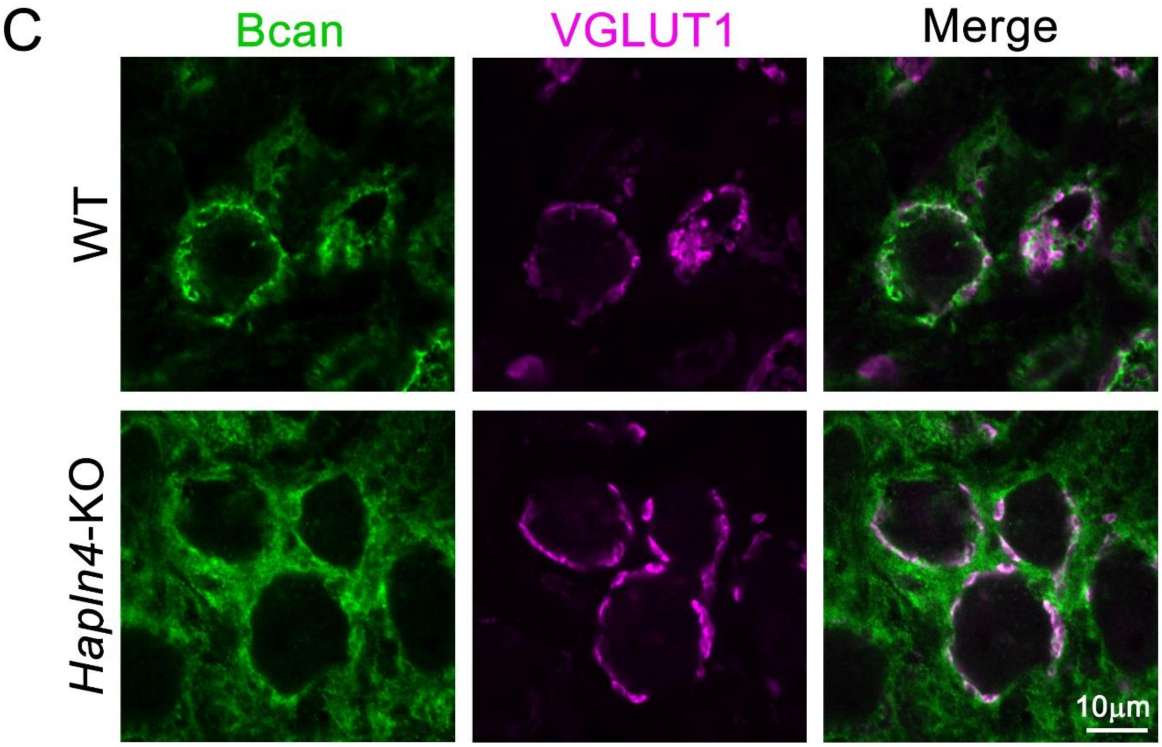

Supplementary Figure 1. Distribution of HAPLN1 and brevican in the adult WT and *Hapln4*-KO mouse MNTB.

Coronal brain sections containing MNTB of 4-month-old *Hapln4*-KO mice and age-matched controls were stained with anti-HAPLN1, anti-brevican, and anti-VGLUT1. (**A, B**) Immunolocalization of HAPLN1 and brevican are compared in the adult WT and *Hapln4*-KO mouse MNTB. The intensity profiles of fluorescence signals along the white lines are shown in the bottom panels. The fluorescence intensities are normalized by the highest intensities of individual proteins within the lines. Of note, colocalized signals are indicated by arrowheads. The well-segregated signals between HAPLN1 and brevican are indicated by arrows (HAPLN1 in green and brevican in magenta, respectively). (**C**) Immunolocalization of brevican (green) is examined in WT and *Hapln4*-KO mouse MNTB using VGLUT1 as a marker of the calyces of Held (magenta). The results are in line with the data shown in Figure 2. Bcan, brevican. Scale bar: 10 μm.

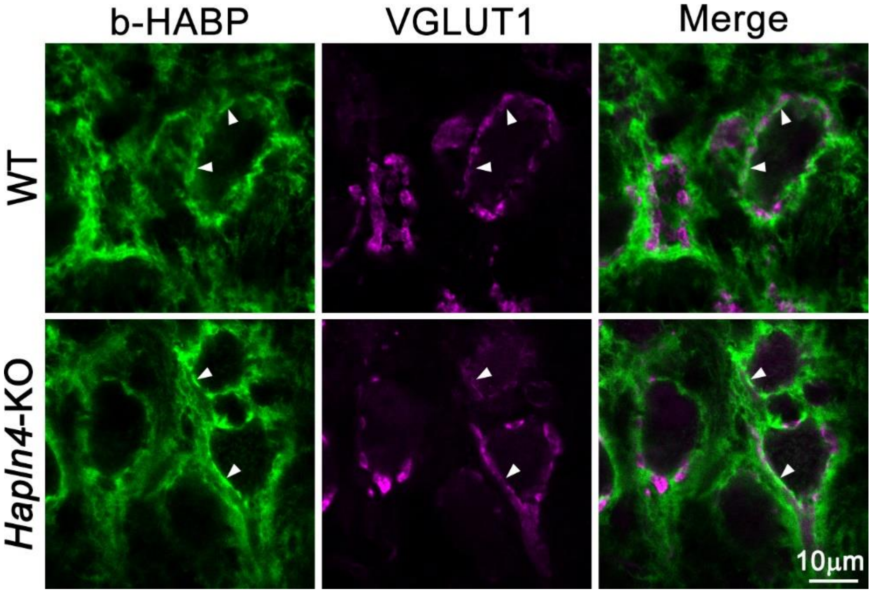

Supplementary Figure 2. Distribution of hyaluronan in the adult mouse MNTB.

Coronal brain sections containing MNTB of 4-month-old *Hapln4*-KO mice and age-matched controls were stained with biotinylated hyaluronan binding protein (b-HABP) (green) and anti-VGLUT1 (magenta). b-HABP signals were found in the inner area of the calyx of Held (arrows). Scale bar: 10 μm.
